# Management of Venous Thromboembolism in Patients with Advanced Gastrointestinal Cancers: What Is the Role of Novel Oral Anticoagulants?

**DOI:** 10.1155/2012/758385

**Published:** 2012-09-11

**Authors:** Ludmila Katherine Martin, Tanios Bekaii-Saab

**Affiliations:** ^1^Division of Medical Oncology, Department of Internal Medicine, The Ohio State University Medical Center, M365 Starling Loving Hall, 410 W 10th Avenue, Columbus, OH 43210, USA; ^2^Division of Medical Oncology, Department of Internal Medicine, The Ohio State University Medical Center, A454 Starling Loving Hall, 410 W 10th Avenue, Columbus, OH 43210, USA

## Abstract

Venous thromboembolism (VTE) is a frequent complication of gastrointestinal cancers that increases morbidity and may impact mortality. Low-molecular-weight heparins (LMWHs) and vitamin K antagonists (VKAs) are standard anticoagulation options for the ambulatory gastrointestinal cancer patient with VTE, but both of these agents are challenging to use for various reasons. Novel oral anticoagulants (NOAs) are new, orally available anticoagulants designed to be easier to administer with more reliable pharmacokinetics that eliminate the need for frequent monitoring of various laboratory parameters. This paper reviews the existing efficacy and safety data for the use of NOAs dabigatran etexilate, rivaroxaban, and apixaban and discusses the potential role of these agents in the management of gastrointestinal cancer-related VTE.

## 1. Introduction

Venous thromboembolism (VTE) is a serious and frequent complication of malignancy. In ambulatory cancer patients, the rate of VTE is reported to be 100-fold higher than the general population [1–3] with an annual incidence of around 1 in 200 [4]. In addition to the underlying hypercoagulable state that accompanies malignancy, cancer patients are at increased risk for VTE for a number of reasons including immobility, surgical procedures, indwelling catheters, and use of chemotherapeutic and antiangiogenic agents that may promote thrombophilia as a side effect. Cancer patients who develop VTE during their disease course appear to be at higher risk of death than those who do not, particularly those in whom VTE is detected at the time of cancer diagnosis [5, 6], however, it is unclear whether this finding is due to consequences of the VTE itself, or merely indicative of more aggressive disease biology.

### 1.1. VTE in Gastrointestinal Cancers

Gastrointestinal malignancies include cancers of the esophagus, stomach, small intestine, appendix, colon, rectum, anus, pancreas, ampulla of Vater, gallbladder, biliary tree, and liver. As a whole, these cancers are associated with a significantly higher incidence of VTE than malignancies of nongastrointestinal origin [7]. In fact, gastrointestinal primary tumor site, and specifically upper gastrointestinal cancer (gastroesophageal and pancreas), has been shown to be independently associated with increased VTE risk in both early-stage malignancy [8] and advanced cancer treated with chemotherapy [7, 14].  Table 1 summarizes the reported rates of VTE in gastrointestinal cancers by tumor site, according to published studies.

In advanced pancreas cancer patients, incidence of VTE is reported as high as 60%, and a recent study found the incidence of VTE to be 50 cases and 20 per 100 person-years in metastatic gastroesophageal and pancreas cancers, with the highest incidence of VTE within 3 months of diagnosis [7]. Multiple studies have suggested an association between VTE and mortality for patients with pancreas cancer [10, 15–22]. A retrospective analysis of the effect of VTE on survival suggested shortened survival from the time of VTE, which varied according to disease site. Patients with pancreas cancer had median survival of 7.5 months from the time of cancer diagnosis and 1.9 months from the time of VTE, while patients with bowel cancer had a median survival of 67 months from diagnosis and 27 months from VTE [23].

In addition to increased incidence of VTE seen in gastrointestinal cancers, therapies for these cancers may exacerbate bleeding risk and promote recurrent VTE. Gastrointestinal cancers are frequently treated with targeted/antiangiogenic therapy (such as bevacizumab, sunitinib, or sorafenib) and chemotherapeutics that commonly induce thrombocytopenia (such as gemcitabine and carboplatin), all of which may increase bleeding risk with anticoagulation. Furthermore, antiangiogenic agents are associated with increased risk of VTE, as is irinotecan [7] and cisplatin [24], both of which are frequently used in the treatment of gastrointestinal cancers. For this high-risk patient population and particularly in those patients with upper gastrointestinal primary tumors, identification of novel, more effective treatment methods that minimize bleeding risk while preventing VTE recurrence are highly important.

### 1.2. Current Standards for VTE Management in the Cancer Patient

Systemic anticoagulation therapy is central to the management of VTE. Goals of therapy include clot stabilization and prevention of clot extension/embolization, avoidance of VTE recurrence, and prevention of long-term complications. Currently available anticoagulants include heparins/low-molecular-weight heparins, indirect factor Xa inhibitors, and vitamin K antagonists (VKAs), all of which have select advantages but significant disadvantages (Table 2). The treatment of malignancy-associated VTE is associated with both benefits and complications. The inconvenience of daily injections and platelet monitoring required with LMWH, or frequent INR monitoring and dose adjustments that accompany VKA therapy, can adversely impact patient quality of life. The concomitant end-organ dysfunction that frequently accompanies malignancy as well as dietary irregularities and malabsorption related to either prior surgery or chemotherapy can make management of anticoagulation with VKAs difficult in this patient population. In addition, chemotherapeutics, targeted agents, and medications used in supportive management all have the potential to interact with anticoagulants. Data suggest that cancer patients with VTE treated with anticoagulation have higher rates of both recurrent VTE (3 times higher) and major bleeding complications (6.5 times higher) than do patients without underlying malignancy, and that this finding cannot be reliably explained by over- or under-anticoagulation alone [10, 11, 14, 25–27].

The randomized CLOT trial [28] established the superiority of LMWH over vitamin K antagonists (VKAs) for reduction in the risk of recurrent cancer-associated VTE. On this study, 672 cancer patients with acute symptomatic VTE received 6 months of either dalteparin or dose-adjusted warfarin. Dalteparin significantly reduced the risk of recurrent VTE with no significant differences in bleeding (major or any) or 6-month overall mortality. In a second randomized trial (CANTHANOX), cancer patients with VTE received initial treatment with enoxaparin, followed bleeding) observed with enoxaparin and a higher incidence of fatal bleeding with warfarin. LMWH has become the standard first-line agents for malignancy-associated VTE, however, by warfarin or further enoxaparin [29]. Although the trial was stopped early due to poor accrual, there were similar incidences of recurrent VTE in both arms but fewer overall events (including their use is often limited by renal dysfunction which is frequently observed in cancer patients, as well as cost and adverse effect of frequent injections on quality of life. Consequently, warfarin is also a reasonable choice for long-term anticoagulation in selecting patients and is frequently used in this setting. While its oral administration is more convenient, the narrow therapeutic window, multitude of dietary and drug-drug interactions, long half-life, and need for frequent monitoring make the administration of warfarin challenging, particularly in the cancer patient.

## 2. Novel Oral Anticoagulants

Current research has focused on the development of novel oral anticoagulants (NOAs) that are safer and easier to administer without compromising efficacy. Ideally, these drugs should be orally administered, have minimal food and drug-drug interactions, wide therapeutic window and predictable pharmacokinetics, no need for monitoring or dose adjustment, low bleeding risk, and rapid onset of action with short half-life. These benefits are particularly relevant to the patient with advanced gastrointestinal cancer. In these patients (provided life expectancy is reasonable), extended anticoagulation is generally recommended over short-course (i.e., 3 months) therapy for VTE due to the high risk of recurrent VTE when anticoagulation is discontinued. Consequently, these patients may be exposed to the risks and inconveniences of anticoagulation for a prolonged period of time, and identification of new agents to mitigate these challenges is very important to their management. This review focuses on NOAs currently in development and their potential role in the management of malignancy-associated VTE in gastrointestinal cancer patients. The oral anticoagulants dabigatran etexilate, rivaroxaban, and apixaban will be discussed in this section. Characteristics of these agents are summarized in Table 3.  Table 4 provides a summary of completed phase III trials of NOAs for the treatment of VTE.

## 3. Dabigatran Etexilate

### 3.1. Background

Dabigatran etexilate (Pradaxa, Boehringer Ingelheim Pharmaceuticals, Ridgefield CT) is an orally available direct thrombin inhibitor with a half-life of 12–14 hours and rapid onset of action (maximum activity is achieved within 2-3 hours) [30]. Bioavailability is relatively poor and an acidic environment is required to facilitate absorption [31, 32]. Excretion is predominantly renal and caution must be used in patients with severe renal impairment [33, 34]. Because of predictable pharmacokinetics, routine monitoring of coagulation parameters is not required. Dabigatran is FDA approved in the United States for VTE prophylaxis following hip and knee arthroplasty, as well as prevention of stroke in patients with nonvalvular atrial fibrillation.

### 3.2. Phase III Clinical Trials

The phase III randomized, double-blind RE-COVER study [35] compared 6 months of dabigatran etexilate 150 mg twice daily (*N* = 1274) to dose-adjusted warfarin (*N* = 1265) for the treatment of acute symptomatic VTE following initial intravenous anticoagulation. The primary endpoint of the study was to demonstrate noninferiority in 6-month incidence of recurrent VTE and VTE-related deaths. 1274 patients were treated and only 10% of the total study population had malignancy-associated DVT. The primary endpoint of noninferiority was met with no difference in the incidence of recurrent VTE (2.4% versus 2.1%, resp.) between the control and experimental arms. There was no difference in major bleeding (1.6% versus 1.9% for experimental and control arms) or treatment-related death. More patients treated with dabigatran experienced adverse events leading to drug discontinuation (9% versus 6.8%), but dyspepsia was the only adverse event observed more frequently with dabigatran (2.9% versus 0.6%). Subgroup analyses of the primary outcome suggested no difference in outcome between patients with and without underlying cancer, and a trend favoring rivaroxaban over warfarin in cancer patients (3.1% with rivaroxaban and 5.3% with warfarin). No subgroup analyses for safety were provided. While hepatotoxicity was rare (*N* = 6 in the total study population) with no differences observed between the groups, 5 of these patients had underlying malignancy (pancreas cancer, *N* = 4, and uterine cancer, *N* = 1). Also of note, inclusion criteria led to a relatively young patient population (median age of 55) that was predominantly Caucasian (>95%) with >90% of patients having creatinine clearance >50 mL/min. A second-phase III trial with the same design, RE-COVER 2 (NCT00681086), has recently been completed with results not yet available.

Two recently presented trials evaluated long-term VTE treatment with dabigatran etexilate. The RE-SONATE study [36] compared 6 months of dabigatran etexilate versus placebo following a 6–18 month course of anticoagulant therapy for long-term treatment of VTE. The primary outcome of recurrent VTE occurred in 0.4% of patients in the experimental group and 5.6% of patients in the control group (HR 0.08) with significantly more clinically relevant bleeding with dabigatran (5.3% versus 1.8%) but no difference in major bleeding. The RE-MEDY trial [36] compared 3–36 months of dabigatran versus warfarin for long-term treatment of VTE following 3–12 months of anticoagulation. The primary outcome of recurrent VTE and related deaths was observed in 1.8% of patients receiving dabigatran and 1.3% of patients receiving warfarin. Dabigatran resulted in significantly less bleeding (19% versus 26%) and also a significant increase in acute coronary syndrome (0.9% versus 0.2%).

## 4. Rivaroxaban

### 4.1. Background

Rivaroxaban (Xarelto, Janssen Pharmaceuticals, Titusville, NJ) is an oral direct inhibitor of factor Xa with excellent bioavailability (80–100%), rapid onset of action (maximum inhibitory activity is reached within 1–4 hours), no significant dietary interactions [37]. Drug-drug interactions are possible however, as rivaroxaban is a substrate of the cytochrome P450 system, particularly CYP3A4. Its half-life is 7–11 hours and it is dually metabolized by both the liver (2/3) and kidney (1/3), therefore, special consideration must be taken in patients with hepatic or renal impairment [38]. No laboratory monitoring is required. Rivaroxaban is currently FDA approved for VTE prophylaxis with hip and knee arthroplasty.

### 4.2. Phase III Clinical Trials

The phase III EINSTEIN-DVT trial [39] randomized patients with acute symptomatic proximal DVT to receive either rivaroxaban 15 mg daily for 3 weeks followed by 20 mg daily for 3, 6, or 12 months (*N* = 1731), or LMWH followed by dose-adjusted VKA (*N* = 1718). Twelve percent of the study population had cancer-related DVT. As in the RE-COVER study, the median age was around 55 years and most patients had normal renal function. The primary endpoint of noninferiority for recurrent VTE was reached with 2.1% of patients in the experimental arm and 3% of patients in the control arm experiencing recurrent VTE (HR 0.68, 95% CI 0.44–1.04, *P* < 0.001). The principal safety outcome of first major or clinically relevant nonmajor bleeding occurred in 8.1% of patients on each arm (*P* = 0.77). There were no differences in adverse events between the two arms. In subgroup analyses, the net clinical benefit (composite of the primary outcome and major bleeding) in patients with active cancer was 3.4% with rivaroxaban and 5.4% with LMWH/VKA, with a hazard ratio favoring rivaroxaban. Subgroup analysis of major and clinically relevant and nonmajor bleeding in cancer patients also favored rivaroxaban (14.4% versus 15.9% with LMWH/VKA). A second similar study, EINSTEIN-PE (NCT00439777), is ongoing to evaluate the efficacy of rivaroxaban in patients with PE with or without DVT.

The EINSTEIN trial also included a parallel double-blind study (EINSTEIN-Extension) evaluating the superiority of continued treatment with rivaroxaban versus placebo in patients who had received 6–12 months of VKA or rivaroxaban for symptomatic DVT or PE [39]. Nine percent of the 1196 patients treated had malignancy-associated VTE. Rivaroxaban was superior to placebo for prevention of recurrent VTE (1.3% versus 7.1%, HR 0.18, *P* < 0.001). There was no difference in major bleeding between the two groups. Subgroup analyses did not include cancer patients.

## 5. Apixaban

### 5.1. Background

Apixaban (Eliquis, Pfizer, New York, NY; Bristol-Myers Squibb, New York, NY) is a direct factor Xa inhibitor that reaches peak concentration 3 hours after oral administration with a half-life of around 12 hours. Oral bioavailability is around 50%> Renal clearance is roughly 25% and metabolism is via the CYP3A4 system [40]. Apixaban is currently not FDA approved for any indication in the United States.

### 5.2. Phase III Clinical Trials

Currently there are 2 phase III trials underway to evaluate apixaban in the treatment of VTE. In the noninferiority AMPLIFY-VTE trial (NCT00643201), patients are randomized to receive either apixaban 10 mg twice daily for 7 days followed by 5 mg twice daily for 6 months or LMWH followed by VKA. The AMPLIFY-EXTENSION trial (NCT00633893) compares 12 months of apixaban to placebo for extended treatment of VTE. Both trials are ongoing.

## 6. Discussion

The ease of administration, lack of required monitoring or dose-adjustments, and minimal food interactions make NOAs attractive agents for the treatment of VTE in the gastrointestinal cancer patient. However, the question remains as to whether these agents will truly simplify the management of VTE in this patient population, and more importantly, whether they are more effective and safer than the current standards of care.

### 6.1. Should NOAs Be Used for the Management of VTE in Gastrointestinal Cancer Patients?

Existing data indicate noninferior therapeutic efficacy and acceptable safety of NOAs in comparison to VKAs and LMWH in the general population. Novel oral anticoagulants, therefore, are promising for the treatment of VTE, however, phase III studies in the acute setting have been noninferiority trials, which are arguably insufficient to change the standard of care. Further investigations are needed to demonstrate superior efficacy, particularly given the lack of significant safety benefit.

There is insufficient data to clearly define the role of NOAs in the gastrointestinal cancer population and further investigation should be undertaken before guidelines can be developed for their use in these patients. Phase III trials have included only small numbers of patients with cancer-related VTE, and there have been no studies specifically investigating the role of these agents for treatment of VTE in the setting of malignancy. Subgroup analyses from existing phase III studies suggest a potential clinical benefit for NOAs in the treatment of cancer-related acute VTE that warrants further investigation, however, the small sample size precludes definitive conclusions and these analyses are exploratory and, therefore, should be interpreted as hypothesis-generating only. In addition, when interpreting the results of these subgroup analyses, it must be kept in mind that these were noninferiority studies, and thus conclusions about superiority cannot be drawn. Strict inclusion criteria of phase III trials limited the number of patients with end-organ dysfunction and resulted in an overall younger (and likely fitter, although performance status was not measured) patient population, which is less representative of the advanced gastrointestinal cancer population and the true efficacy and safety of these agents in these patients may not be adequately assessed. Finally, LMWH is generally considered superior to VKA in the treatment of malignancy-associated VTE, and future studies should evaluate the efficacy of NOAs in direct comparison with LMWH, rather than VKAs, in the cancer population.

### 6.2. Are NOAs Safe in Patients with Gastrointestinal Cancers?

Several potential safety concerns specific to the gastrointestinal cancer population (and cancer patients as a whole) exist with NOAs that have not been extensively investigated. The cancer population is known to have a higher risk of bleeding while on standard anticoagulant therapy [14, 25]. While the more reliable pharmacokinetics of NOAs may partially mitigate this risk, bleeding complications of NOAs are not well defined in this patient population who at baseline may be predisposed to hemorrhage for multiple other reasons including chemotherapy-induced thrombocytopenia or receipt of antiangiogenic therapy. In addition, concomitant liver or kidney dysfunction (discussed further below) may decrease clearance of NOAs in these patients, thereby potentially increasing bleeding risk. Subgroup analyses from the EINSTEIN-DVT trial suggest that rivaroxaban may be associated with a slightly lower risk of bleeding than LMWH/VKA in cancer patients, but this needs to be further prospectively investigated in a larger population.

The potential for drug-drug interactions with NOAs may be higher in patients with advanced gastrointestinal cancers than in the general population. NOAs are substrates of either the CYP (rivaroxaban and apixaban) or p-glycoprotein (dabigatran and apixaban) systems. The CYP system, particularly CYP3A4, is involved in the metabolism of oral chemotherapeutic and targeted agents used in the treatment of gastrointestinal cancers (such as capecitabine, which can induce CYP2D9, and sorafenib, sunitinib, imatinib, and erlotinib). P-glycoprotein is a drug efflux pump which is implicated in multidrug chemotherapy resistance. Medications frequently used in the supportive care of gastrointestinal cancer patients (such as steroids, antibiotics, antidepressants, or antiemetics) can induce or inhibit either CYP3A4 or p-glycoprotein, and result in drug interactions with NOAs which may result in either increased toxicity or decreased therapeutic efficacy.

Patients with severe liver and kidney disease have generally been excluded from clinical trials of NOAs. Liver impairment is frequently seen in gastrointestinal cancer patients, either as a result of metastatic disease (the liver is the most common site of metastases for gastrointestinal cancers), biliary obstruction, or chemotherapy-related liver toxicity. Although hepatotoxicity was not frequently observed on any phase III study of NOAs, on the RE-COVER study, 4 of the 6 patients experiencing liver injury (and 1 of the 2 patients receiving dabigatran) had underlying advanced pancreas cancer, indicating that caution may be required in these patients who are already predisposed to hepatotoxicity, however, additional investigation is necessary to further characterize the potential risk of liver injury. Additionally, rivaroxaban and dabigatran are partially hepatically cleared, and their use may be limited in the gastrointestinal cancer population which frequently has underlying liver dysfunction. Patients with hepatocellular carcinoma commonly have underlying cirrhosis and the use of rivaroxaban would also be limited in these patients, as it is contraindicated in Child's class B and C cirrhosis.

NOAs are all renally cleared to a degree, with rivaroxaban and dabigatran being the most extensively eliminated in the urine. These agents are both contraindicated in patients with severely impaired renal function. Increased trough concentrations of dabigatran have been observed with even mild renal impairment, and dabigatran exposure (AUC) is 2.7-fold higher in moderate renal insufficiency [33]. In patients with gastrointestinal cancer, renal dysfunction can result from anticancer treatment (directly, as with platinum agents, or indirectly, as with dehydration from chemotherapy-induced gastrointestinal toxicity), disease, or other comorbidities. As with LMWH, careful monitoring of renal function as well as assessment for bleeding would be necessary during the use of NOAs in these patients.

The oral administration of NOAs may be more convenient than injectable anticoagulants, however, this may also be problematic for the gastrointestinal cancer patient. These patients have frequently undergone prior surgeries which may alter the anatomy of the gastrointestinal tract or may have chemotherapy-related gastrointestinal toxicity, both of which can potentially affect the absorption of medications. This is particularly concerning with dabigatran, which already has very limited bioavailability. While pharmacokinetic studies of NOAs account for weight differences [33, 41], the effect of nutritional status on the pharmacokinetics and pharmacodynamics of these agents is less well studied. Gastrointestinal cancer patients, particularly those with biliary and pancreas cancers, frequently suffer from cancer cachexia, a syndrome of characterized by anorexia, malnutrition, and loss of lean muscle and fat stores. Loss of circulating proteins and albumins is frequently seen in the later stages of this syndrome, and, it could potentially affect the binding and levels of NOAs, which are variably protein bound (rivaroxaban 95%, dabigatran 35%). Although previous studies have concluded that interactions involving protein binding may not be clinically relevant for these agents [33], further investigation is likely required.

Finally, gastrointestinal cancers tend to have a median age of onset between 65–75 years of age. Pharmacokinetic studies suggest 40–60% increased exposure of dabigatran in older versus younger patients [33], likely relating to age-related decline in renal function. The potential implication of this finding on the risk of bleeding and other drug toxicities in cancer patients is unclear.

## 7. Conclusions

In conclusion, although NOAs are promising for the treatment of VTE in the general population, they should not be used as first-line therapy for VTE in patients with advanced gastrointestinal cancers outside the setting of a clinical trial, as there is insufficient data to clearly define their efficacy and safety. LMWH should remain standard first-line therapy for cancer-related DVT. Prospective investigations of NOAs specific to the cancer population are necessary.

## Figures and Tables

**Table 1:** Incidence of VTE in patients with gastrointestinal cancers.

Primary site	VTE incidence (per 100 person-years/% of patients)
Gastroesophageal [6–9]	50/7–14
Pancreas [6–10]	20/5–60
Colorectal and anal [7–9, 11]	13.7/3–10
Hepatobiliary [7, 9, 12, 13]	4.6/2–15

**Table 2:** Characteristics of currently available anticoagulants.

	Unfractionated heparin	Low molecular weight heparin	Fondaparinux	VKA
	Target antithrombin	Factor Xa, IIa (variable)	Xa	Factors II, VII, IX, X

Advantages	(1) Rapid onset of action	(1) Rapid onset of action	(1) Rapid onset of action	(1) Orally administered
	(2) Reversible	(2) Partially reversible	(2) No monitoring required	(2) Reversible
	(3) Can be used in renal dysfunction	(3) No drug-drug interactions	(3) Fixed dose	(3) Can be used in renal dysfunction
	(4) No drug-drug interactions	(4) No monitoring required		
		(5) Fixed dose		

	(1) Requires monitoring (platelet, PTT)	(1) Heparin-induced thrombocytopenia*	(1) Frequent injections	(1) Requires monitoring (INR)
	(2) Frequent injections	(2) Cannot be used in severe renal dysfunction	(2) No antidote	(2) Drug-drug interactions
	(3) Heparin-induced thrombocytopenia	(3) Frequent injections	(3) Cannot be used in severe renal dysfunction	(3) Food interactions
Disadvantages	(4) Requires dose adjustment	(4) Bleeding risk	(4) Bleeding risk	(4) Narrow therapeutic window
	(5) Bleeding risk			(5) Delayed onset of action/cannot be used alone in acute VTE
	(6) Risk of osteoporosis			(6) Requires dose adjustment
				(7) Bleeding risk

*Lower risk than unfractionated heparin.

**Table 3:** Characteristics of novel oral anticoagulants.

Agent	Trade name	Target	Bioavailability (%)	Time to peak concentration (h)	Half-Life (h)	Excretion	Dosing frequency	Drug interactions	Contraindications	Monitoring?
Dabigatran	Pradaxa	IIa	3–7	2-3	12–14	Urine (80%) Biliary (20%)	BID	P-glycoprotein	CrCl < 30 mL/min	No
Rivaroxaban	Xarelto	Xa	80	2.4–4	5–9	Urine (66%), Biliary (28%)	Daily	CYP-3A4	CrCl < 30 mL/min Hemodialysis Child-Pugh B, C	No
Apixaban	Eliquis	Xa	50	3	8–15	Urine (25%)	Daily	CYP-3A4 P-glycoprotein	CrCl < 15 mL/min	No

**Table 4:** Summary of completed phase III trials of novel oral anticoagulants.

Study	Agent investigated	Control	Setting	Treatment duration	N/N with cancer-related VTE	Primary outcome	HR (95% CI)	*P* value	VTE recurrence	Clinically relevant bleeding
RE-COVER	Dabigatran etexilate*	Warfarin*	Acute symptomatic VTE	6 months	2539/121	Non-inferiority	1.1 (0.65, 1.84)	<0.001	E: 2.4% C: 2.1%	E: 5.6% C: 8.8%
RE-SONATE^a^	Dabigatran etexilate	Placebo	Treated VTE	6 months	1343/NA**	Superiority	0.08 (0.02, 0.25)	<0.0001	E: 0.4% C: 5.6%	E: 5.3% C: 1.8%
RE-MEDY^a^	Dabigatran etexilate	Warfarin	Treated VTE	6–36 months	2856/NA	Non-inferiority	1.44 (0.78, 2.64)	0.03	E: 1.8% C: 1.3%	NA** NA**
EINSTEIN-DVT	Rivaroxaban	LMWH/VKA	Acute DVT	3, 6 or 12 months	3449/207	Non-inferiority	0.68 (0.44, 1.04)	<0.001	E: 2.1% C: 3%	E: 8.1% C: 8.1%
EINSTEIN-Extension	Rivaroxaban	Placebo	Treated VTE	6 or 12 months	1196/54	Superiority	0.18 (0.09, 0.39)	<0.001	E: 1.3% C: 7.1%	E: 6% C: 1.2%

*Following initial treatment with intravenous anticoagulation.

**Not available.

*Abstract only.

## References

[1] Cushman M, Tsai AW, White RH (2004). Deep vein thrombosis and pulmonary embolism in two cohorts: the longitudinal investigation of thromboembolism etiology. *American Journal of Medicine*.

[2] Heit JA, Michael O’Fallon W, Petterson TM (2002). Relative impact of risk factors for deep vein thrombosis and pulmonary embolism: a population-based study. *Archives of Internal Medicine*.

[3] Naess IA, Christiansen SC, Romundstad P, Cannegieter SC, Rosendaal FR, Hammerstrøm J (2007). Incidence and mortality of venous thrombosis: a population-based study. *Journal of Thrombosis and Haemostasis*.

[4] Lee AY, Levine MN (2003). Venous thromboembolism and cancer: risks and outcomes. *Circulation*.

[5] Sørensen HT, Mellemkjaer L, Olsen JH, Baron JA (2000). Prognosis of cancers associated with venous thromboembolism. *The New England Journal of Medicine*.

[6] Chew HK, Wun T, Harvey D, Zhou H, White RH (2006). Incidence of venous thromboembolism and its effect on survival among patients with common cancers. *Archives of Internal Medicine*.

[7] Shah MA, Capanu M, Soff G, Asmis T, Kelsen DP (2010). Risk factors for developing a new venous thromboembolism in ambulatory patients with non-hematologic malignancies and impact on survival for gastroesophageal malignancies. *Journal of Thrombosis and Haemostasis*.

[8] Khorana A, Kuderer NM, Culakova E, Lyman GH, Francis CW (2008). Development and validation of a predictive model for chemotherapy- associated thrombosis. *Blood*.

[9] Wun T, White RH (2009). Venous thromboembolism (VTE) in patients with cancer: epidemiology and risk factors. *Cancer Investigation*.

[10] Epstein AS, Soff GA, Capanu M (2012). Analysis of incidence and clinical outcomes in patients with thromboembolic events and invasive exocrine pancreatic cancer. *Cancer*.

[11] Hutten BA, Prins MH, Gent M, Ginsberg J, Tijssen JGP, Buller HR (2000). Incidence of recurrent thromboembolic and bleeding complications among patients with venous thromboembolism in relation to both malignancy and achieved International Normalized Ratio: a retrospective analysis. *Journal of Clinical Oncology*.

[12] Jeon HK, Kim DU, Baek DH (2012). Venous thromboembolism in patients with cholangiocarcinoma: focus on risk factors and impact on survival. *European Journal of Gastroenterology & Hepatology*.

[13] Connolly GC, Chen R, Hyrien O (2008). Incidence, risk factors and consequences of portal vein and systemic thromboses in hepatocellular carcinoma. *Thrombosis Research*.

[14] Sallah S, Wan JY, Nguyen NP (2002). Venous thrombosis in patients with solid tumors: determination of frequency and characteristics. *Thrombosis and Haemostasis*.

[15] Menapace LA, Peterson DR, Berry A, Sousou T, Khorana AA (2011). Symptomatic and incidental thromboembolism are both associated with mortality in pancreatic cancer. *Thrombosis and Haemostasis*.

[16] Mandalà M, Tondini C (2011). The impact of thromboprophylaxis on cancer survival: focus on pancreatic cancer. *Expert Review of Anticancer Therapy*.

[17] Mikal S, Campbell AJA (1950). Carcinoma of the pancreas. Diagnostic and operative criteria based on one hundred consecutive autopsies. *Surgery*.

[18] Miller JR, Baggenstross AH, Comfort MW (1951). Carcinoma of the pancreas; effect of histological types and grade of malignancy on its behavior. *Cancer*.

[19] Sack GH, Levin J, Bell WR (1977). Trousseau’s syndrome and other manifestations of chronic disseminated coagulopathy in patients with neoplasms: clinical, pathophysiologic, and therapeutic features. *Medicine*.

[20] Sgouros J, Maraveyas A (2008). Excess premature (3-month) mortality in advanced pancreatic cancer could be related to fatal vascular thromboembolic events. A hypothesis based on a systematic review of phase III chemotherapy studies in advanced pancreatic cancer. *Acta Oncologica*.

[21] Thodiyil PA, Kakkar AK (2002). Variation in relative risk of venous thromboembolism in different cancers. *Thrombosis and Haemostasis*.

[22] Moore MJ, Goldstein D, Hamm J (2007). Erlotinib plus gemcitabine compared with gemcitabine alone in patients with advanced pancreatic cancer: a phase III trial of the National Cancer Institute of Canada Clinical Trials Group. *Journal of Clinical Oncology*.

[23] Prestidge T, Lee S, Harper P, Young L, Ockelford P (2012). Survival in patients with malignancy and venous thromboembolism by tumour subtype and thrombus location. *Journal of Internal Medicine*.

[24] Moore RA, Adel N, Riedel E (2011). High incidence of thromboembolic events in patients treated with cisplatin-based chemotherapy: a large retrospective analysis. *Journal of Clinical Oncology*.

[25] Prandoni P, Lensing AWA, Piccioli A (2002). Recurrent venous thromboembolism and bleeding complications during anticoagulant treatment in patients with cancer and venous thrombosis. *Blood*.

[26] Alcalay A, Wun T, Khatri V (2006). Venous thromboembolism in patients with colorectal cancer: incidence and effect on survival. *Journal of Clinical Oncology*.

[27] Douketis JD, Crowther MA, Foster GA, Ginsberg JS (2001). Does the location of thrombosis determine the risk of disease recurrence in patients with proximal deep vein thrombosis?. *The American Journal of Medicine*.

[28] Lee AY, Levine MN, Baker RI (2003). Low-molecular-weight heparin versus a coumarin for the prevention of recurrent venous thromboembolism in patients with cancer. *The New England Journal of Medicine*.

[29] Meyer G, Marjanovic Z, Valcke J (2002). Comparison of low-molecular-weight heparin and warfarin for the secondary prevention of venous thromboembolism in patients with cancer: a randomized controlled study. *Archives of Internal Medicine*.

[30] Van Ryn J, Stangier J, Haertter S (2010). Dabigatran etexilate—a novel, reversible, oral direct thrombin inhibitor: interpretation of coagulation assays and reversal of anticoagulant activity. *Thrombosis and Haemostasis*.

[31] Hankey GJ, Eikelboom JW (2011). Dabigatran etexilate: a new oral thrombin inhibitor. *Circulation*.

[32] Tran A, Cheng-Lai A (2011). Dabigatran etexilate: the first oral anticoagulant available in the united states since warfarin. *Cardiology in Review*.

[33] Stangier J, Clemens A (2009). Pharmacology, pharmacokinetics, and pharmacodynamics of dabigatran etexilate, an oral direct thrombin inhibitor. *Clinical and Applied Thrombosis/Hemostasis*.

[34] Stangier J, Rathgen K, Sthle H, Mazur D (2010). Influence of renal impairment on the pharmacokinetics and pharmacodynamics of oral dabigatran etexilate: an open-label, parallel-group, single-centre study. *Clinical Pharmacokinetics*.

[35] Schulman S, Kearon C, Kakkar AK (2009). Dabigatran versus warfarin in the treatment of acute venous thromboembolism. *The New England Journal of Medicine*.

[36] Schulman S, Baanstra D, Eriksson H (2011). Dabigatran versus placebo for extended maintenance therapy of venous thromboembolism. *Journal of Thrombosis and Haemostasis*.

[37] Kubitza D, Becka M, Voith B, Zuehlsdorf M, Wensing G (2005). Safety, pharmacodynamics, and pharmacokinetics of single doses of BAY 59-7939, an oral, direct factor Xa inhibitor. *Clinical Pharmacology and Therapeutics*.

[38] Kubitz D, Becka M, Roth A, Mueck W (2008). Dose-escalation study of the pharmacokinetics and pharmacodynamics of rivaroxaban in healthy elderly subjects. *Current Medical Research and Opinion*.

[39] Bauersachs R, Berkowitz SD, Brenner B (2010). Oral rivaroxaban for symptomatic venous thromboembolism. *The New England Journal of Medicine*.

[40] Raghavan N, Frost CE, Yu Z (2009). Apixaban metabolism and pharmacokinetics after oral administration to humans. *Drug Metabolism and Disposition*.

[41] Kreutz R (2012). Pharmacodynamic and pharmacokinetic basics of rivaroxaban. *Fundamental & Clinical Pharmacology*.

